# Dietary oregano essential oil supplementation improves intestinal functions and alters gut microbiota in late-phase laying hens

**DOI:** 10.1186/s40104-021-00600-3

**Published:** 2021-07-06

**Authors:** Jia Feng, Mingyuan Lu, Jing Wang, Haijun Zhang, Kai Qiu, Guanghai Qi, Shugeng Wu

**Affiliations:** Laboratory of Quality & Safety Risk Assessment for Animal Products on Feed Hazards (Beijing) of the Ministry of Agriculture & Rural Affairs, Feed Research Institute, Chinese Academy of Agricultural Sciences, No. 12 Zhongguancun South St., Haidian District, Beijing, 10081 China

**Keywords:** Essential oil, Feed efficiency, Immune status, Late-phase laying hen, Microbiota

## Abstract

**Background:**

Dietary essential oil (EO) supplementation can exert favorable effects on gut health in broilers. However, it is unknown whether EO could improve intestinal functions, consequently beneficial for egg performance and quality in late-phase laying hens. This study was aimed to investigate the potential effects of EO on production performance, egg quality, intestinal health and ileal microbiota of hens in the late phase of production. A total of 288 60-week-old Hy-line Brown laying hens were randomly divided into 4 groups and fed a basal diet (control) or basal diets supplemented with oregano EO at 100, 200 and 400 mg/kg (EO100, EO200 and EO400).

**Results:**

Dietary EO supplementation resulted in a quadratic decrease (*P* < 0.05) in feed conversion ratio with lower (*P* < 0.05) feed conversion ratio in EO200 group than the control during weeks 9–12 and 1–12 of the trial. Compared to the control, EO addition resulted in higher (*P* < 0.05) eggshell thickness at the end of week. 4, 8 and 12 and higher (*P* < 0.05) chymotrypsin activity. There was a quadratic elevation (*P* < 0.05) in ileal chymotrypsin and lipase activity, along with a linear increase in villus height to crypt depth ratio. Quadratic declines (*P* < 0.05) in mRNA expression of *IL-1β*, *TNF-α*, *IFN-γ* and *TLR-4*, concurrent with a linear and quadratic increase (*P* < 0.05) in *ZO-1* expression were identified in the ileum with EO addition. These favorable effects were maximized at medium dosage (200 mg/kg) of EO addition and intestinal microbial composition in the control and EO200 groups were assessed. Dietary EO addition increased (*P* < 0.05) the abundances of Burkholderiales, Actinobacteria, Bifidobacteriales, Enterococcaceae and Bacillaceae, whereas decreased *Shigella* abundance in the ileum.

**Conclusions:**

Dietary EO addition could enhance digestive enzyme activity, improve gut morphology, epithelial barrier functions and modulate mucosal immune status by altering microbial composition, thus favoring feed efficiency and eggshell quality of late-phase laying hens.

## Introduction

Declined laying performance and poor egg quality in the late laying period have seriously reduced the economic benefits, which are the key obstacles for extending laying period especially in late-phase laying hens [1]. The compromised intestinal functions, immune imbalance and intestinal flora disturbance due to high-intensity production are ascribed for the poor egg performance of hens in late laying period [2, 3]. Owing to the restriction of antibiotic utilization as growth promoters for animals, the use of natural bioactive compounds such as essential oils (EO) has attracted much attention to improve poultry health and performance [4].

EO are aromatic oily liquids extracted from plant material (flowers, buds, seeds, leaves, etc.), whose antibacterial properties have encouraged their usage as natural antibiotic alternatives for animal production [4]. The efficacy of EO on reducing the colonization of *Escherichia coli*, *Clostridium perfringens* and *Campylobacter jejuni* have been extensively investigated in broiler [5, 6] and pig [7, 8]. EO or their main components (such as thymol and carvacrol) can disturb the membrane structure and alter its permeability by partitioning the lipid fraction of plasma membrane, and thus exert antibacterial activity [4]. Furthermore, the beneficial effects of EO have been widely documented in poultry production. The supplementation of EO (containing thymol) in broiler diets may enhance growth performance, increase intestinal and pancreatic digestive enzyme activities [9, 10] and improve cellular and humoral immunity [11, 12]. A combined use of thymol and carvacrol was demonstrated to alleviate intestinal inflammation, the impaired intestinal integrity and barrier dysfunction induced by *C. perfringens* challenge in broilers [13]. Besides directly inhibiting the growth of pathogen bacteria, EO (blends of thymol and carvacrol or encapsulated cinnamaldehyde) could modulate intestinal microbial composition of birds [14, 15]. Thus, EO may exert a favorable effect on gut health via maintaining intestinal integrity and barrier functions, enhancing immune system activities and regulating gut microbiota. The inclusion of EO (thymol, carvacrol or menthol as active components) in layer diets was reported to improve laying performance and egg quality [16–18]. However, the effects of EO on the intestinal microbial community, mucosal barrier and immune status of laying hens in the late phase of production await further studies.

A great variety of plants are valued for their EO content and oregano (*Origanum vulgare L.*) are among the most widely used [4]. The main compounds of oregano EO are thymol, carvacrol, γ-terpinene and *p*-cimene, which exhibits some biological activities including antibacterial, anti-inflammatory and immune-regulating properties [7, 8]. In the present study, we hypothesized that dietary inclusion of oregano EO would positively alter the microbial composition, mucosal immune responses, and intestinal barrier functions, subsequently conducing to the improvements of egg performance and quality in laying hens. Therefore, this study was aimed to investigate the effects of dietary EO supplementation on laying performance, egg quality, gut morphology, microbial community, and relative mRNA expression of immune-related and tight junction-related genes in the intestine of laying hens in the late phase of production.

## Materials and methods

### Birds and experimental design

The animal protocols for this study were approved by the Animal Care and Use Committee of the Feed Research Institute of the Chinese Academy of Agricultural Sciences. A total of 288 60-week-old Hy-line Brown laying hens were randomly divided into 4 groups with 8 replicates of 9 birds each. The layers were commercially purchased from Xiaoming Agriculture and Animal Husbandry Co. Ltd. (Ningxia, China). Prior to the experiment, egg production and egg quality were assessed, which were similar across all the replicates. Birds received the basal diet without (Control) or with 100 mg/kg (EO100), 200 mg/kg (EO200) and 400 mg/kg (EO400) microencapsulated oregano EO product for 12 weeks. Oregano EO product (containing 5% oregano EO) was obtained from a commercial supply with carvacrol and thymol as active ingredients and calcium carbonate as carrier. The designed concentrations of oregano EO in the diets of EO100, EO200 and EO400 groups were 5, 10 and 20 mg/kg, respectively. The concentration of carvacrol and thymol in EO determined by high-performance liquid chromatography (HPLC) was ≥2.83% and ≥ 0.08%, respectively. All birds were housed in three-tier battery cages with 3 birds per cage (cage size: 45 cm × 45 cm × 45 cm) in an environmentally controlled house with temperature maintained at approximately 24 °C. All birds were fed with the same corn-soybean meal basal diet and provided with feed and water *ad libitum* with exposure to 16 h of light/d. The basal diet (Table 1) was formulated according to NRC (1994) recommendations. All hens remained in good health and medical intervention was not applied to any birds during the whole feeding period.

**Table 1:** Ingredient and nutrient levels of the experimental diets (air-dried basis)

Ingredients, %		Nutrient levels, %^b^	
Corn	61.00	Metabolizable energy, MJ/kg	11.27
Soybean meal	23.86	Crude protein	16.50
Soybean oil	1.20	Calcium	3.47
Wheat bran	3.35	Available phosphorus	0.27
NaCl	0.15	Lysine	0.81
Na_2_SO_4_	0.20	Methionine	0.37
CaHPO_4_	0.90	Methionine+cystine	0.65
Limestone	8.90		
Premix^a^	0.20		
Choline	0.12		
DL-Methionine	0.12		
Total	100.00		

^a^ Premix provided the following per kg of the diet: vitamin A, 12,500 IU; vitamin D_3_, 4125 IU; vitamin E, 15 IU; vitamin K, 2 mg; thiamine, 1 mg; riboflavin, 8.5 mg; calcium pantothenate, 11 mg; niacin, 32.5 mg; pyridoxine, 8 mg; biotin, 0.5 mg; folic acid, 1.25 mg; vitamin B_12_, 0.02 mg; Mn, 65 mg; I, 1 mg; Fe, 60 mg; Cu, 8 mg; Zn, 66 mg; phytase, 500 mg

^b^ The nutrient levels were calculated values

### Sample collection

Five eggs per replicate were collected for egg quality determination every 4 weeks. Besides, one bird from each replicate was randomly selected at the end of weeks. 12 of the experiment and the intestinal tract was separated after slaughter. The middle portion of ileum were then isolated and approximately 1 cm segments of the midpoints of ileum were fixed in 10% neutral-buffered formalin for histological analysis. Ileal digesta were snap-frozen in liquid nitrogen and stored at − 80 °C for analysis of digestive enzyme activity and microbial composition. The mucosa samples of ileum were collected by gentle scraping the intestinal wall with glass microscope slides, snap-frozen in liquid nitrogen and stored at − 80 °C for mRNA analysis.

### Laying performance and egg quality

Egg production and egg weight were recorded daily by replicate and feed consumption for each replicate was weighed every 4 weeks. Feed conversion ratio (FCR) was calculated as grams of feed consumption/egg weight for each replicate. Average daily feed intake and FCR were calculated every 4 weeks. As for egg quality measurement, each egg was individually weighed and egg height (mm) and width (mm) were recorded for shape index calculation (shape index = height/width). Breaking strength and the thickness of eggshell were measured by Egg Force Reader and Egg Shell Thickness Gauge (Israel Orka Food Technology Ltd., Ramat Hasharon, Israel). Haugh unit values, albumen height, and yolk color were measured by an Egg Analyzer (Israel Orka Food Technology Ltd., Ramat Hasharon, Israel). The eggshells were cleaned, air-dried, and weighed. Relative eggshell weight was calculated as eggshell weight/egg weight × 100.

### Intestinal morphological analysis

Ileal tissues fixed in formalin were embedded in paraffin and paraffin sections (5 μm) were sliced using a microtome and mounted on glass slides. The sections were dewaxed with xylene, hydrated, and then stained with hematoxylin and eosin (H and E). For each sample, three intact villi-crypt units were selected for morphology observation using a light microscope coupled with image-processing software (Image J 1.53). Villus height (VH, the height from the tip of villus to the villus-crypt junction) and crypt depth (CD, the depth of invagination between adjacent villi) were measured. VH to CD ratio (VCR) was calculated.

### Digestive enzyme activity of ileal digesta

The activities of amylase, lipase, and chymotrypsin in intestinal digesta from the ileum were determined by colorimetry using assay kits (Nanjing Jiancheng Bioengineering Institute of China, Nanjing, China).

### RNA isolation and real-time quantitative PCR

Total RNA was extracted from the ileum mucosa using EasyPureTM RNA kit (Beijing Transgene Biotech Ltd., Beijing, China) following the manufacturer’s instructions. The purity and concentration of the total RNA were measured by Epoch Microplate Spectrophotometer (BioTek Instruments, Inc., VT, USA). The cDNA samples were obtained by reverse transcription of the total RNA using the first-strand synthesis kit (TransGen Biotech Co., Ltd., Beijing, China). Real-time PCR for analysis of the gene expression was performed using SYBR Green (Thermo Fisher Scientific, MA, USA) on an ABI 6 flex real-time PCR instrument (Thermo Fisher Scientific, MA, USA). Primer sequences used in this study are shown in Table 2. The reaction conditions were as follows: 50 °C for 2 min, 95 °C for 10 min; 40 cycles of 95 °C for 15 s, 60 °C for 1 min. Melt curve analysis was performed to confirm the PCR amplification specificity. Each sample was measured in duplicate and the relative mRNA expression levels were analyzed using β-actin as an internal control by the 2^-ΔΔCt^ method [19].

**Table 2:** Sequences of real-time PCR primers

Genes	Primer sequence (5′→3′)	Accession no.
Claudin-1	F: AAGTGCATGGAGGATGACCA	NM_001013611.2
	R: GCCACTCTGTTGCCATACCA	
Occludin	F: TCATCGCCTCCATCGTCTAC	NM_205128.1
	R: TCTTACTGCGCGTCTTCTGG	
*ZO-1*	F: TATGAAGATCGTGCGCCTCC	XM_015278981.1
	R: GAGGTCTGCCATCGTAGCTC	
Mucin-2	F: AGCGAGATGTTGGCGATGAT	NM_001318434.1
	R: AAGTTGCCACACAGACCACA	
*IL-1β*	F: ACTGGGCATCAAGGGCTA	NM_204524
	R: GGTAGAAGATGAAGCGGGTC	
*IL-8*	F: GGCTTGCTAGGGGAAATGA	AJ009800
	R: AGCTGACTCTGACTAGGAAACTGT	
*IL-10*	F: CGCTGTCACCGCTTCTTCA	NM_001004414.2
	R: CGTCTCCTTGATCTGCTTGATG	
*TNF-α*	F: GAGCGTTGACTTGGCTGTC	NM_204267
	R: AAGCAACAACCAGCTATGCAC	
*IFN-γ*	F: AAAGCCGCACATCAAACACA	NM_205149.1
	R: GCCATCAGGAAGGTTGTTTTTC	
*TLR-4*	F: CCACTATTCGGTTGGTGGAC	NM_001030693.1
	R: ACAGCTTCTCAGCAGGCAAT	
β-actin	F: GAGAAATTGTGCGTGACATCA	L08165
	R: CCTGAACCTCTCATTGCCA	

*F* forward primer, *R* reverse primer

*ZO-1* zonula occludens-1, *IL* interleukin, *TNF-α* tumor necrosis factor-α, *IFN-γ* interferon-γ, *TLR* toll-like receptors

### DNA extraction and analysis of ileal microbiota

The gut digesta samples (~ 200 mg of each sample) were used for microbial DNA extraction using QIAamp DNA Stool Mini Kit (Qiagen, Hilden, Germany) according to the manufacturer’s instructions. The quality of DNA samples was assessed by 1% agarose gel electrophoresis. The V3-V4 region of the 16S rRNA gene was amplified using the primer pair 338F/806R (5′-ACTCCTACGGGAGGCAGCA-3′ and 5′-GGACTACHVGGGTWTCTAAT-3′). PCR products were quantified with the PicoGreen dsDNA Assay Kit (Invitrogen, Carlsbad, USA). The Illumina platform was used to generate paired-end reads (2 × 300 bp). Sequencing and bioinformatics were performed on QIIME2 platform of Shanghai Personal Biotechnology Co., Ltd. (Shanghai, China) and the sequencing results were analyzed based on amplicon sequence variants (ASVs) [20]. Two obvious outliers in each group may interfere with the microbiota statistical analysis and thus were excluded in the following analysis. Alpha diversity indices (including Chao1 richness estimator, Observed_species, Shannon diversity index, and Simpson index) were calculated to evaluate microbial species evenness. Beta diversity was evaluated by principal coordinate analysis (PCoA) based on the unweighted UniFrac distance. Taxa abundances at the phylum, class, order, family and genus levels were statistically compared between groups. Linear discriminant analysis (LDA) combined effect size measurements (LEfSe) were used to identify the differences in microbial composition between groups. Pearson correlation analysis was conducted on the potential relationship between ileal gene expression levels, digestive enzyme activity, gut morphology, performance parameter and microbial composition.

The sequencing data have been deposited at the National Center of Biotechnology Information (NCBI) Sequence Read Archive (SRA) database (accession number: PRJNA693086).

### Statistical analysis

Data were analyzed by one-way Analysis of Variance (ANOVA) procedure and differences were examined using Duncan’s Multiple Range Test using SAS Version 9.2 (SAS Institute Inc., Cary, NC, USA). The linear and quadratic effects of dietary EO supplementation dose were evaluated by regression analysis. The differences in the relative abundances of bacteria between groups were assessed using Wilcoxon rank tests. Data were presented as mean with their pool standard error of the mean (SEM) or mean ± standard deviation and statistical significance was defined as a *P* value < 0.05.

The regression model was as follows:

$$ \mathrm{Y}\ \mathrm{ij}=\upalpha +\upbeta 1\mathrm{Xi}+\mathrm{eij}\ \left(\mathrm{linear}\ \mathrm{regression}\right),\mathrm{Y}\ \mathrm{ij}=\upalpha +\upbeta 1\mathrm{Xi}+\upbeta 2\mathrm{Xi}2+\mathrm{eij}\ \left(\mathrm{quadratic}\ \mathrm{regression}\right). $$

Yij was the response variable; α was the intercept (indicators with the basal diet); β1 and β2 were regression coefficient; Xi was the studied factor effect as the inclusion of EO (i = 0, 100, 200, 400), and eij was the observational error for (ij)th observation.

## Results

### Laying performance and egg quality

Dietary EO supplementation had no significant influences (*P* > 0.05) on egg production and average daily feed intake of laying hens during weeks 1–4, 5–8, 9–12 and weeks 1–12 of the experiment (Table 3). However, average egg weight increased (*P* < 0.05) linearly with the elevated levels of EO in diets during weeks 1–4, 5–8 and 1–12. There was a quadratic decrease (*P* ≤ 0.05) in FCR with the increasing addition of EO during the whole experiment period. During weeks 9–12 and 1–12, dietary EO supplementation at 100 mg/kg decreased (*P* ≤ 0.05) FCR of laying hens in comparison with the control. With regard to egg quality, there was no significant effects (*P* > 0.05) of dietary EO supplementation on eggshell strength, relative eggshell weight, shape index, albumen height, Haugh unit and yolk color at the end of week 4, 8 and 12 (Table 4). Eggshell thickness increased linearly and quadratically (*P* < 0.05) in response to the increasing addition of EO and eggshell thickness in EO supplemented groups were higher (*P* < 0.05) than that in the control at the end of week 4, 8 and 12.

**Table 3:** Effects of dietary supplementation with essential oil on laying performance of laying hens^1^

Items	Treatments^2^				SEM^3^	*P*-value		
	Control	EO100	EO200	EO400		ANOVA	Linear	Quadratic
Egg production								
weeks 1–4	0.880	0.905	0.907	0.905	0.0055	0.243	0.174	0.138
weeks 5–8	0.863	0.898	0.894	0.893	0.0064	0.183	0.197	0.134
weeks 9–12	0.854	0.861	0.885	0.861	0.0060	0.282	0.598	0.233
weeks 1–12	0.865	0.888	0.896	0.888	0.0050	0.144	0.163	0.064
Average egg weight, g								
weeks 1–4	60.51	60.27	61.13	61.85	0.27	0.147	0.031	0.092
weeks 5–8	60.65	60.24	61.31	62.03	0.26	0.079	0.017	0.052
weeks 9–12	60.47	60.08	60.99	61.36	0.24	0.240	0.083	0.216
weeks 1–12	60.55	60.19	61.14	61.75	0.24	0.110	0.028	0.083
Average daily feed intake, g/hen per day								
weeks 1–4	111.66	111.28	108.77	111.97	0.68	0.331	0.948	0.304
weeks 5–8	109.47	110.97	108.64	111.02	0.66	0.524	0.592	0.768
weeks 9–12	108.30	109.72	110.57	110.74	0.72	0.631	0.244	0.415
weeks 1–12	110.33	110.26	108.68	111.08	0.54	0.467	0.683	0.411
Feed conversion ratio, g/g								
weeks 1–4	2.10	2.05	1.96	2.00	0.021	0.075	0.064	0.039
weeks 5–8	2.10	2.06	1.99	2.01	0.018	0.236	0.053	0.050
weeks 9–12	2.12^a^	2.10^a^	2.01^b^	2.09^ab^	0.015	0.029	0.272	0.048
weeks 1–12	2.11^a^	2.07^ab^	1.98^b^	2.03^ab^	0.016	0.026	0.053	0.017

^1^ *n* = 8 replicates per treatment

^2^ Control, hens received a basal diet; EO100, EO200 and EO400, hens received a basal diet supplemented with 100, 200 or 400 mg/kg oregano essential oil, respectively

^3^ SEM, standard error of the mean

^a-b^ Values within a row with no common superscripts differ significantly (*P* < 0.05)

**Table 4:** Effects of dietary supplementation with essential oil on egg quality of laying hens^1^

Items	Treatments^2^				SEM^3^	*P*-value		
	Control	EO100	EO200	EO400		ANOVA	Linear	Quadratic
Eggshell thickness, 10^−2^ mm								
week 4	42.31^b^	44.19^a^	44.28^a^	44.69^a^	0.23	0.002	0.002	0.001
week 8	42.86^b^	44.21^a^	44.39^a^	44.54^a^	0.18	0.001	0.002	< 0.001
week 12	42.25 ^b^	44.00^a^	44.10^a^	44.56^a^	0.20	< 0.001	< 0.001	< 0.001
Eggshell strength, N								
week 4	35.51	37.02	36.95	36.72	0.32	0.322	0.314	0.217
week 8	34.24	35.63	35.10	34.58	0.38	0.804	0.980	0.536
week 12	34.00	34.89	35.65	35.84	0.39	0.330	0.089	0.176
Relative eggshell weight, %								
week 4	9.43	9.67	9.45	9.55	0.058	0.430	0.795	0.915
week 8	9.26	9.52	9.44	9.57	0.051	0.135	0.065	0.136
week 12	9.26	9.68	9.55	9.62	0.063	0.075	0.125	0.107
Shape index								
week 4	1.35	1.34	1.35	1.35	0.0039	0.950	0.968	0.886
week 8	1.35	1.34	1.35	1,34	0.0031	0.541	0.424	0.546
week 12	1.37	1.36	1.37	1.36	0.0026	0.374	0.106	0.261
Albumen height, mm								
week 4	6.10	6.32	6.44	6.37	0.11	0.744	0.440	0.533
week 8	6.46	6.87	6.84	6.74	0.081	0.275	0.400	0.185
week 12	7.07	7.30	7.31	7.27	0.080	0.716	0.504	0.532
Haugh unit								
week 4	75.88	77.51	77.93	77.36	0.89	0.873	0.628	0.705
week 8	79.08	82.21	81.38	80.32	0.59	0.266	0.772	0.231
week 12	82.93	84.74	82.17	83.94	0.67	0.564	0.859	0.959
Yolk color								
week 4	5.65	5.83	5.60	5.90	0.11	0.735	0.512	0.760
week 8	5.45	5.80	5.63	5.93	0.080	0.167	0.071	0.197
week 12	4.83	4.90	4.78	4.85	0.064	0.927	0.974	0.990

^1^ n = 8 replicates per treatment

^2^ Control, hens received a basal diet; EO100, EO200 and EO400, hens received a basal diet supplemented with 100, 200 or 400 mg/kg oregano essential oil, respectively

^3^ SEM, standard error of the mean

^a-b^ Values within a row with no common superscripts differ significantly (*P* < 0.05)

### Intestinal morphology and digestive enzyme activity

No significant changes (*P* > 0.05) were observed in ileal VH and CD of laying hens at the end of week 12 with the increasing EO supplementation, which, however, linearly elevated (*P* < 0.05) ileal VCR (Table 5). EO supplementation did not affect (*P* > 0.05) α-amylase activity in ileal digesta (Table 6). However, there was a quadratic elevation (*P* < 0.05) in ileal chymotrypsin activity along with a linear and quadratic increase (*P* < 0.05) in ileal lipase activity. Compared with the control, dietary EO inclusion at the level of 100 mg/kg increased (*P* < 0.05) ileal chymotrypsin activity.

**Table 5:** Effects of dietary supplementation with essential oil on ileal morphology of laying hens^a^

Items^b^	Treatments^c^				SEM^d^	*P*-value		
	Control	EO100	EO200	EO400		ANOVA	Linear	Quadratic
VH, μm	587.61	609.70	731.26	585.89	25.92	0.145	0.922	0.153
CD, μm	149.58	144.35	161.19	125.41	5.67	0.156	0.159	0.143
VCR	4.02	4.23	4.60	4.64	0.11	0.128	0.029	0.064

^a^ *n* = 8 replicates per treatment at the end of week 12

^b^ VH, villus height; CD, crypt depth; VCR, villus height to crypt depth ratio

^c^ Control, hens received a basal diet; EO100, EO200 and EO400, hens received a basal diet supplemented with 100, 200 or 400 mg/kg oregano essential oil, respectively

^d^ SEM, standard error of the mean

**Table 6:** Effects of dietary supplementation with essential oil on digestive enzyme activity (U/g) of ileal digesta^1^

Items	Treatments^2^				SEM^3^	*P*-value		
	Control	EO100	EO200	EO400		ANOVA	Linear	Quadratic
Chymotrypsin	495.68^c^	556.43^b^	617.16^a^	531.75^bc^	10.60	< 0.001	0.300	< 0.001
α-amylase	561.93	573.45	533.00	578.53	10.58	0.444	0.737	0.574
Lipase	2.97	3.25	4.21	4.10	0.20	0.059	0.023	0.040

^1^ *n* = 8 replicates per treatment at the end of week 12

^2^ Control, hens received a basal diet; EO100, EO200 and EO400, hens received a basal diet supplemented with 100, 200 or 400 mg/kg oregano essential oil, respectively

^3^ SEM, standard error of the mean

^a-b^ Values within a row with no common superscripts differ significantly (*P* < 0.05)

### Gene expression in the ileal tissue

**Fig. 1:**
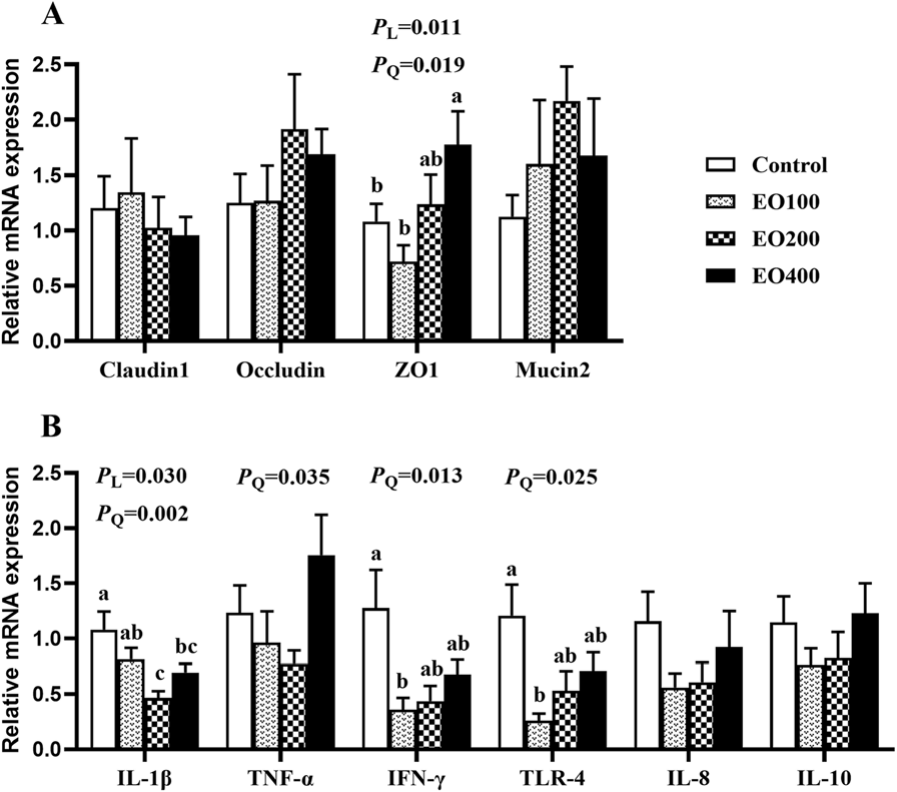
Effects of dietary supplementation with essential oil on the relative mRNA expression of genes. **a** and **b** were results of relative mRNA expression of genes related to tight junction proteins and immune response, respectively. Data are expressed as means ± standard deviation. ^a-c^ Treatments with no common superscripts differ significantly (*p* < 0.05). Control, hens received a basal diet; EO100, EO200 and EO400, hens received a basal diet supplemented with 100, 200 or 400 mg/kg oregano essential oil, respectively. L and Q represent the linear and quadratic effects of dietary essential oil supplementation dose assessed by regression analysis. ZO1: Zonula occludens 1; IL: Interleukin; TNF-α: Tumor necrosis factor-α; IFN-γ: Interferon-γ; TLR: Toll-like receptors

No significant influences (*P* > 0.05) were observed on relative mRNA expression of claudin-1, occludin and mucin-2 in ileal mucosa of laying hens in response to the addition of EO (Fig. 1a). However, there was a linear and quadratic increase (*P* < 0.05) in relative mRNA expression of zonula occludens-1 (*ZO-1*) in the ileum with the increasing levels of EO in diets. Dietary supplementation with EO quadratically down-regulated (*P* < 0.05) the relative mRNA expression of interleukin-1β (*IL-1β*), tumor necrosis factor-α (*TNF-α*), interferon-γ (*IFN-γ*) and toll-like receptor-4 (*TLR-4*) in the ileum by the increasing addition of EO (Fig. 1b).

### Ileal microbial profile

**Fig. 2:**
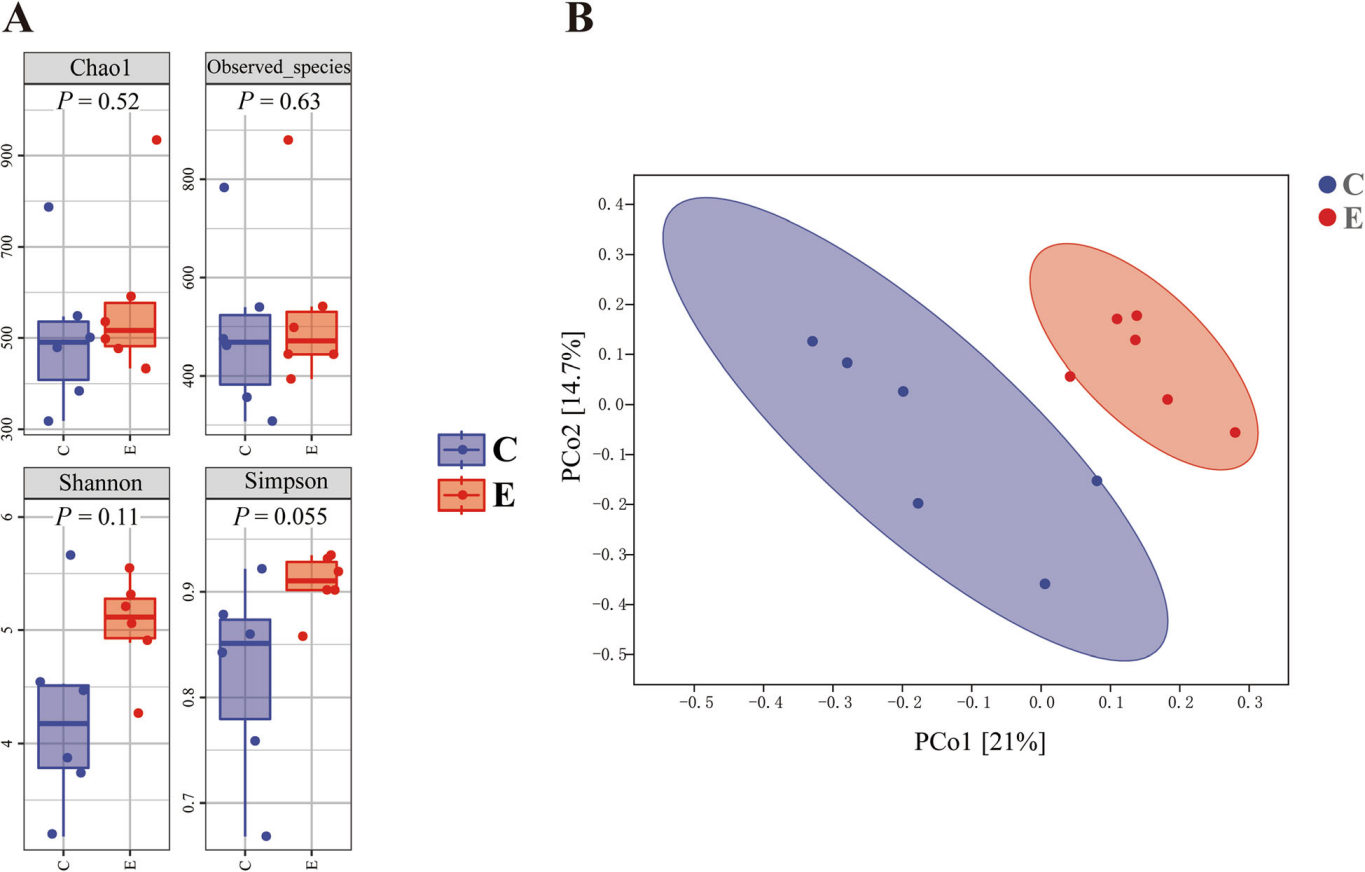
Alpha (**a**) and Beta (**b**) diversity analysis of ileal microbiota from laying hens. Beta diversity analysis with principal coordinates analysis (PCoA) was based on the unweighted UniFrac distance. **c**, control; **e**, essential oil-supplemented group (control + essential oil addition at 200 mg/kg)

**Fig. 3:**
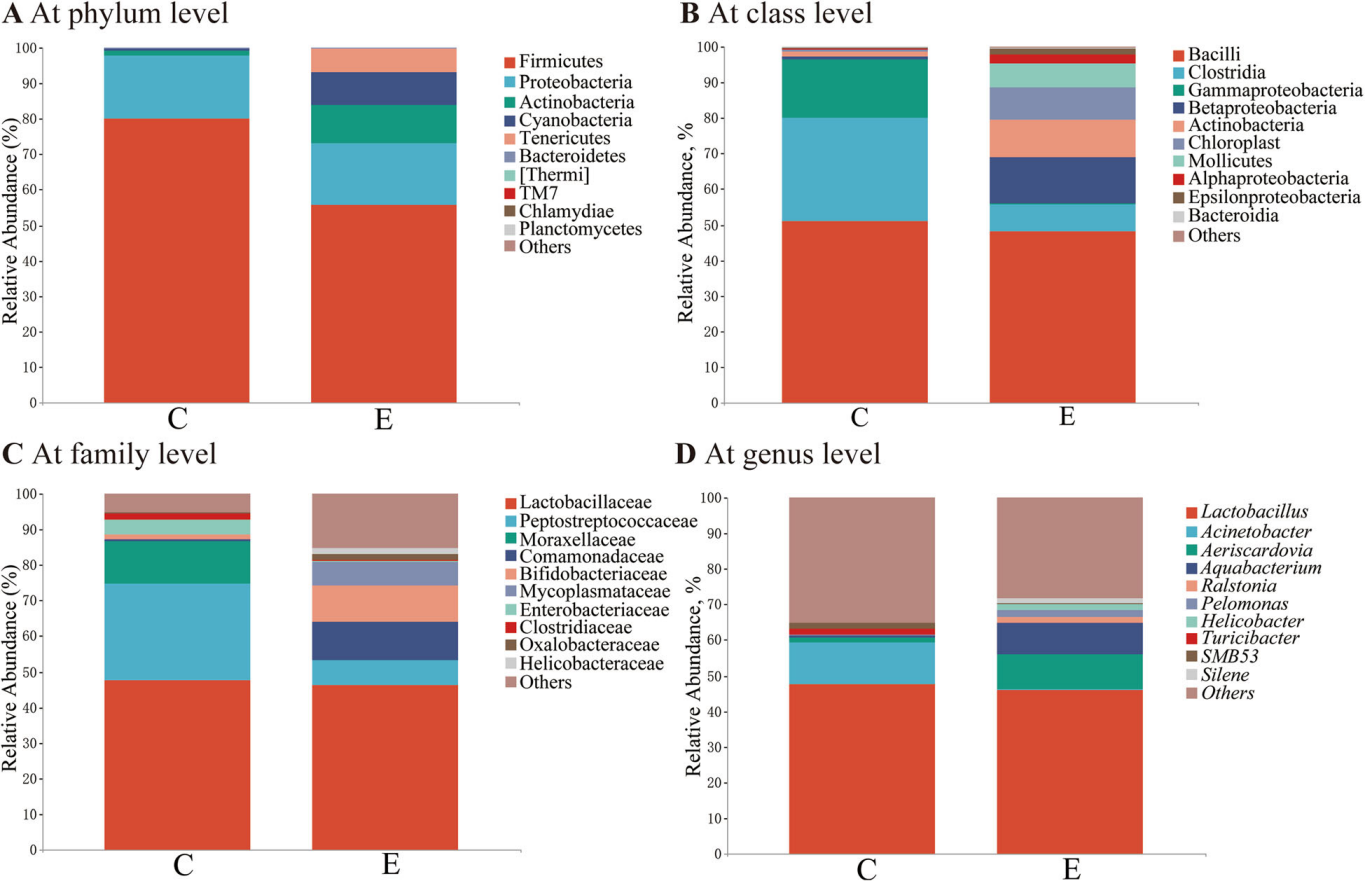
Relative abundance of ileal microbiota from laying hens. **a** at phylum level, **b** at class level, **c** at family level, and **d** at genus level. **c**, control; **e**, essential oil-supplemented group (essential oil addition at 200 mg/kg)

No significant differences (*P* > 0.05) in species richness (as reflected by Chao1 and Observed_species indices) or alpha-diversity (as reflected by Shannon and Simpson indices) were observed in ileal microbiota at the taxonomic level (Fig. 2a). However, PCoA results based on the unweighted UniFrac distance showed separation of ileal microbial communities between control and EO-supplemented groups (Fig. 2b). Firmicutes and Proteobacteria were the dominant phyla in the control and EO-supplemented groups, account for more than 70% of the whole phyla (Fig. 3a). EO supplementation resulted in a decreased abundance of Firmicutes. The dominant classes were Bacilli and Clostridia, within Firmicutes across groups (Fig. 3b). Family analysis indicated that the abundance of Bifidobacteriaceae tended to increase (*P* = 0.060; Fig. 3c) with EO addition. At genus level, the *Lactobacillus* accounted for the greatest proportion of the microbiota (Fig. 3d). The abundance of *Aeriscardovia* and *Aquabacterium* abundances were increased (*P* = 0.064 and 0.018) with EO addition.

**Fig. 4:**
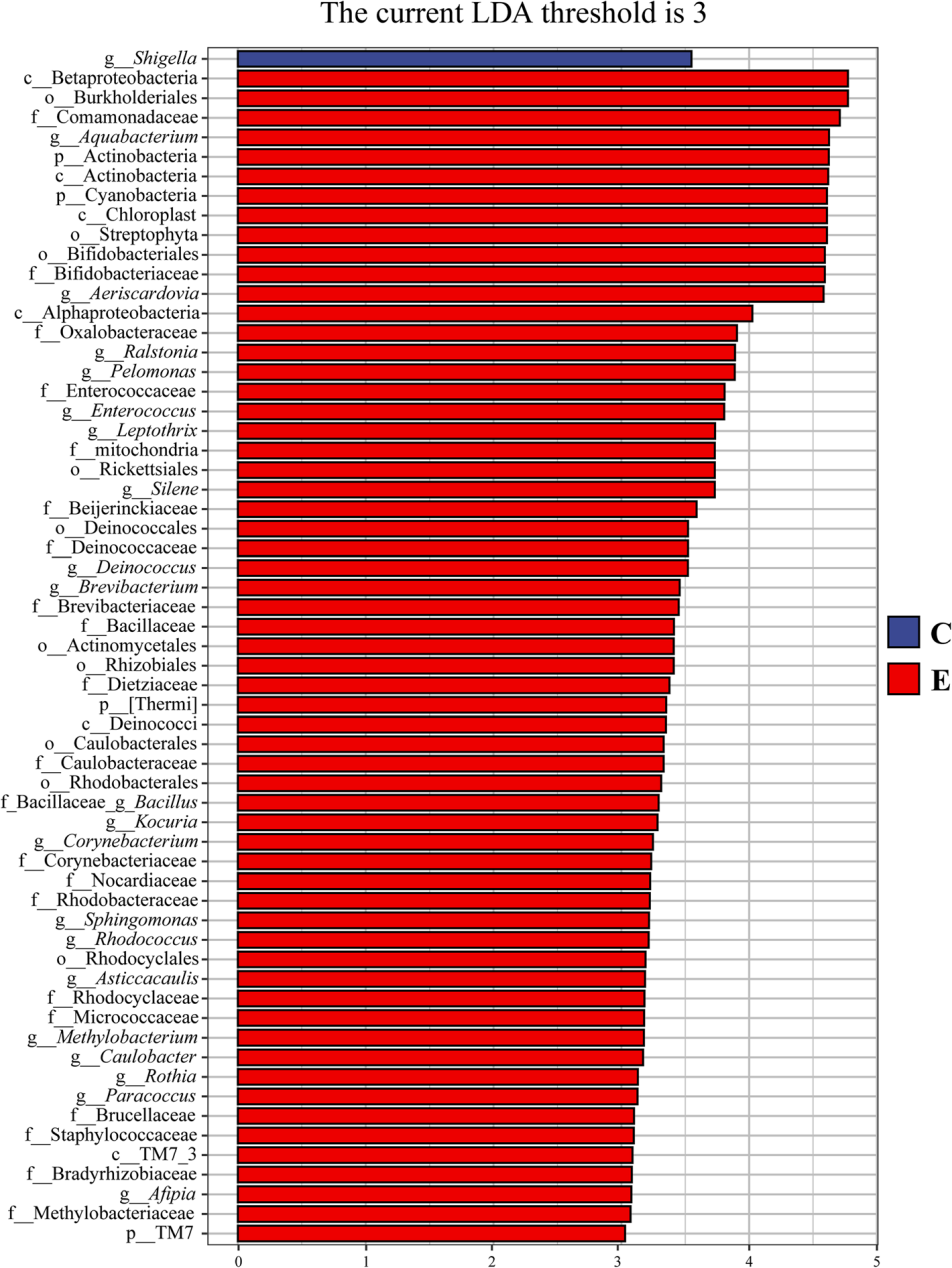
Linear discriminant analysis (LDA) combined effect size measurements (LEfSe) analysis of ileal microbiota. C, control; E, essential oil-supplemented group (essential oil addition at 200 mg/kg). Species with significant difference that have an LDA score greater than the estimated value (3.0). The length of the histogram represents the LDA score

The LEfSe analysis was conducted to identify the relative richness (*P* < 0.05, LDA > 3.0; Fig. 4) of bacterial members in the ileum of two groups. *Shigella* was found to be enriched in the control, while the microbiota in EO-supplemented group was differentially enriched with Burkholderiales, Actinobacteria, Bifidobacteriales, Enterococcaceae, Bacillaceae, *Kocuria* and Corynebacteriaceae. Compared with the control, dietary inclusion of EO increased (*P* < 0.05) the abundances of Cyanobacteria, Burkholderiales and Comamonadaceae and simultaneously tended to increase (*P* < 0.1) Actinbacteria (Actinobacteria) and Bifidobacteriales (Bifidobacteriaceae) abundances (Table 7).

**Table 7:** Differences of bacterial distribution in ileal digesta between the control and essential oil supplementation groups^a^

Items, %	C^b^	E	*P*-value
**Phyla**			
Actinobacteria	1.48 ± 2.86	10.68 ± 8.88	0.052
Cyanobacteria	0.63 ± 0.81	9.13 ± 7.66	0.042
**Classes**			
Betaproteobacteria	0.74 ± 0.79	12.77 ± 8.71	0.019
Actinobacteria	1.48 ± 2.85	10.59 ± 8.83	0.053
Chloroplast	0.63 ± 0.81	9.12 ± 7.66	0.042
Alphaproteobacteria	0.32 ± 0.39	2.53 ± 1.12	0.004
**Orders**			
Burkholderiales	0.73 ± 0.79	12.76 ± 8.70	0.019
Bifidobacteriales	1.43 ± 2.80	10.04 ± 8.68	0.060
Streptophyta	0.63 ± 0.81	9.12 ± 7.66	0.042
**Families**			
Comamonadaceae	0.60 ± 0.66	10.97 ± 7.42	0.019
Bifidobacteriaceae	1.43 ± 2.80	10.04 ± 8.68	0.060
Oxalobacteraceae	0.12 ± 0.12	1.77 ± 1.33	0.013
**Genera**			
*Aeriscardovia*	1.41 ± 2.80	9.86 ± 8.69	0.064
*Aquabacterium*	0.40 ± 0.54	8.89 ± 6.00	0.018
*Ralstonia*	0.10 ± 0.10	1.69 ± 1.34	0.033
*Pelomonas*	0.07 ± 0.05	1.69 ± 1.46	0.023
*SMB53*	1.56 ± 1.61	0.18 ± 0.28	0.090
*Silene*	0.23 ± 0.30	1.39 ± 1.06	0.027

^a^ Data are represented with the means ± standard deviation (*n* = 6)

^b^ C, control; E, essential oil-supplemented group (essential oil addition at 200 mg/kg)

### Correlation between ileal microbiota and mucosal gene expression, production performance, digestive enzyme activity or gut morphology

**Fig. 5:**
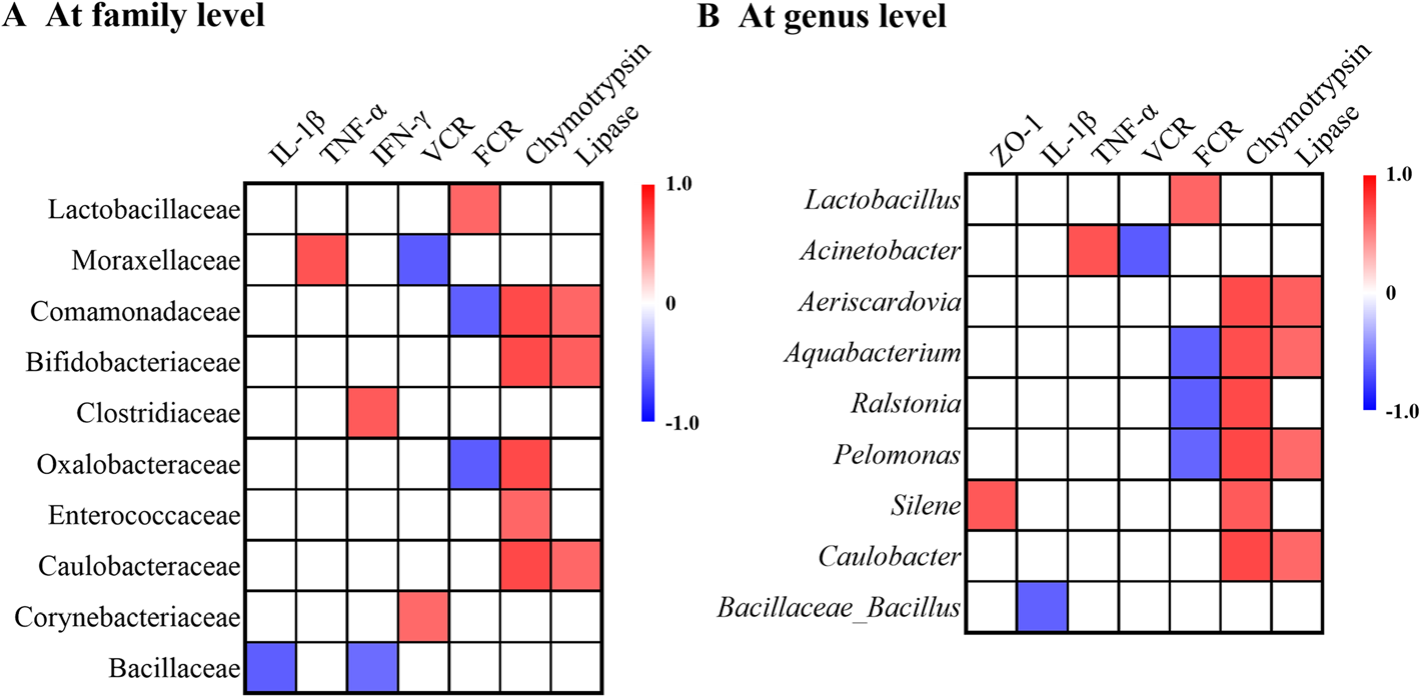
Pearson’s correlation analysis between the abundances of ileal microbiota and production or intestinal parameters. **a** at family level and **b** at genus level. The intensity of the colors represents the degree of association. Red represents a significant positive correlation (*P* < 0.05), blue represents significantly negative correlation (*P* < 0.05), and white shows that the correlation was not significant (*P* > 0.05)

A Pearson correlation analysis was employed to determine whether there was any association among laying performance, intestinal morphology, digestive enzyme activity, mucosal gene expression and main bacterial members. Correlation analysis revealed that the mRNA expression of *IL-1β* and *TNF-α* was negatively correlated (*P* < 0.05) with the abundances of Bacillaceae, whereas *TNF-α* and *IFN-γ* expression were positively correlated (*P* < 0.05; Fig. 5a) with Moraxellaceae and Clostridiaceae abundances, respectively. VCR was negatively correlated (*P* < 0.05) with Moraxellaceae, but it had a positive correlation (*P* < 0.05) with Corynebacteriaceae. FCR showed a positive correlation (*P* < 0.05) with Lactobacillaceae, while showed a negative correlation (*P* < 0.05) with Comamonadaceae and Oxalobacteraceae. There was a positive correlation (*P* < 0.05) between the activity of chymotrypsin and the abundances of Comamonadaceae, Bifidobacteriaceae, Oxalobacteraceae, Enterococcaceae and Caulobacteraceae, and a negative correlation (*P* < 0.05) between the activity of lipase and Comamonadaceae, Bifidobacteriaceae and Caulobacteraceae abundances. At genus level, the mRNA expression of *ZO-1* was positively correlated (*P* < 0.05; Fig. 5b) with *Silene* abundance, but *IL-1β* expression was negatively correlated (*P* < 0.05) with the number of *Bacillaceae_Bacillus*. The abundance of *Acinetobacter* was positively correlated (*P* < 0.05) with *TNF-α* expression, but was negatively correlated (*P* < 0.05) with VCR. FCR showed a negative correlation (*P* < 0.05) with the abundances of *Aquabacterium*, *Ralstonia* and *Pelomonas*, but showed a positive correlation (*P* < 0.05) with *Lactobacillus* abundance. The abundances of *Aeriscardovia*, *Aquabacterium*, *Pelomonas* and *Caulobacter* were positively correlated (*P* < 0.05) with lipase and chymotrypsin activity, and chymotrypsin activity was positively correlated (*P* < 0.05) with *Ralstonia* and *Silene* abundances.

## Discussion

In the present study, dietary EO supplementation improved feed efficiency but showed no statistical effects on egg production of late-phase hens. Consistent with our findings, several recent studies have indicated that EO improved the feed utilization in broilers [21, 22] and laying hens [18, 23]. These beneficial effects could be attributed to the active components (thymol and carvacrol) in EO, which have been demonstrated to exhibit antimicrobial, anti-inflammation activities as well as improvements in gut health status and utilization of nutrients [4, 24]. It may be of great importance to alleviate age-related deleterious effects on intestinal health and functions, since intestinal health problems were regarded as a crucial reason for poor laying performance in late-phase hens [3, 25]. In this study, the increased VCR and digestive enzyme activities, improved immune homeostasis and altered microbiota structure suggested an enhancement of digestion and absorption function and a healthy condition of intestine, thus benefiting feed utilization of laying hens. In contrast, other studies suggested that EO or their main compounds yielded no significant improvements in terms of laying performance of laying hens [26, 27]. The inconsistencies in the efficacy of EO on production performance may be related to the composition and supplemental levels of EO, the basal diet, bird age, and the environmental conditions. Poor physiological conditions of late-phase laying hens in this study may favor the efficacy of EO on feed utilization. Interestingly, in this study, feed efficiency was not affected by EO addition at a higher supplemental level (400 mg/kg). It was reported that carvacrol or thymol at high concentrations might exert negative effects on intestinal epithelial cells and gut beneficial bacteria [28, 29], which may subsequently compromise feed utilization of laying hens. It was speculated that dietary EO supplementation exhibited favorable effects on feed utilization at an appropriate level (200 mg/kg), whereas these improvements may be masked by adverse effects at a higher concentration (400 mg/kg). However, the double-edged effects of EO on intestinal functions and gut microbiota need further investigation. In this study, the medium dose (200 mg/kg) was the most effective in terms of production performance and egg quality of laying hens.

It is well known that digestive enzymes, including chymotrypsin, α-amylase and lipase, are involved in nutrient digestion for absorption and their activities, which are crucial for feed utilization and production performance of animals. It has been reported that EO could stimulate digestive secretions such as bile acids, gastric and digestive enzymes (e.g., lipase, amylase and proteases) in rats [30]. Similarly, the use of phytogenic products containing thymol, carvacrol or other active components in the diets of broilers and pigs elevated the activities of intestinal amylase, protease, and lipase [10]. In this study, there was an improvement in the activities of ileal chymotrypsin and lipase in response to the increased EO addition. It could be postulated that dietary EO supplementation could enhance the digestive enzyme activities, possibly resulting in the accelerated digestion of protein and fat and thus assisting with their absorption in the intestine. The increased digestive enzyme activities might be due to the antibacterial activity of thymol and carvacrol, and their modulatory effects on intestinal microbial composition [4], resulting in less pathogen-induced damage of enterocytes. It would further diminish the risk of pathogen invasion to intestinal epithelial cells and promote their ability to regenerate villus. Mature villus cells implied they are more active to secret enzymes than immature crypt cells [31]. This was supported by the findings that the addition of EO, with thymol or carvacrol as active compounds, exhibited positive effects on intestinal morphology, evidenced by increased VH and VCR accompanied with decreased CD [6, 13, 17]. Likewise, in this study, there was a linear increase in VCR with the increased EO addition, indicating an enhancement of absorptive surface area, efficient enzyme secretion and nutrient transport, possibly benefiting nutrient utilization in the intestine. The declined absorption efficiency of intestinal calcium has been recognized as the main cause for the poor eggshell quality in late-phase laying hens [32, 33]. In this study, the enhanced villi-crypts absorptive area following EO addition could be favorable to calcium absorption [34], resulting in an elevation in calcium deposition into eggshell along with increased eggshell thickness. Another potential mechanism reported was that EO could improve uterine health and an appropriate site would be provided for eggshell calcification, consequently increasing eggshell weight and thickness [18]. Thus, the improvements of eggshell quality in response to EO supplementation may be attributed to the beneficial effects of dietary EO addition on intestinal and uterine health, whereas the underlying mechanisms need to be further studied.

Disruption of tight junctions and microbiota dysbiosis due to long-term egg production would enable the translocation of luminal pathogens and toxins [35, 36]. It would subsequently lead to inflammation and tissue damage, which may be partially responsible for the lower nutrient absorption and the compromised laying performance of laying hens in the late production period. In this study, EO addition down-regulated mRNA expression of *TLR-4* and pro-inflammatory cytokines *IL-1β*, *TNF-α* and *IFN-γ*, while the expression of anti-inflammatory cytokines in the ileum was not affected, which were consistent with previous studies in broilers [13, 22]. TLRs, the core components in mucosal innate immune responses, can recognize microbiota and their products and finally initiate inflammatory responses with the release of pro-inflammatory cytokines [37]. TLR-4 is involved in the recognition of lipopolysaccharide (LPS), a unique component of the outer membrane of gram-negative bacteria such as *Escherichia coli*, *Salmonella* and *Shigella* strains. The decreased expression of *TLR-4*, *IL-1β*, *TNF-α* and *IFN-γ* might be associated with the antimicrobial properties of EO, suggesting that EO could exert anti-inflammatory activity in the ileum of laying hens, possibly by suppressing the activation of TLR4-mediated signaling pathway. In fact, anti-inflammatory activities of thymol and carvacrol have been well documented. They can suppress the expression of proinflammatory cytokines, stimulate the expression of anti-inflammatory cytokines, prevent inflammatory cell recruitment and thus attenuate inflammation [38, 39]. However, in this study, the expression of anti-inflammatory cytokines was not affected by EO treatment and the exact mechanism of anti-inflammatory activity of EO in laying hens required further investigation. Additionally, TLRs are also involved in the regulation of intestinal barrier integrity [40]. Pro-inflammatory cytokines, such as IL-1β, TNF-α and IFN-γ, can activate NF-κB signaling pathway and then impair the epithelial barrier function by dysregulating tight junctions [41, 42]. ZO-1, one of the tight junction proteins, is an essential component of intestinal barrier and plays a crucial role in regulating intestinal permeability and integrity [43]. In the present study, oregano EO supplementation upregulated the mRNA expression of intestinal barrier gene *ZO-1* in the ileum compared to that in the control, which may be associated with the suppressed expression of pro-inflammatory cytokines. It indicated that oregano EO could improve the barrier function of intestinal epithelium and consequently strengthen immune defense against pathogen infection. Similarly, dietary inclusion of thymol or carvacrol was reported to upregulate the mRNA expression of occludin, *ZO-1* and claudin-1 in small intestine of broiler chickens regardless of *C. perfingens* challenge [6, 13]. Thymol treatment could enhance the barrier function of epithelial cells by increasing the protein level of ZO-1 in the IPEC-J2 cell model [44]. Therefore, the improved immune status and strengthened epithelial barrier in response to EO treatment would be beneficial for the maintenance of gut health and production performance of laying hens.

To better understand the favorable effects of EO, further analysis was conducted on gut microbiota, whose interactions with gut play a crucial role in prevention of pathogen colonization, maintenance of immune homeostasis and metabolism of nutrient. Since the favorable effects of EO supplementation were mainly observed in EO200 group, the modulatory roles of EO on intestinal microbial composition were assessed in the control and EO200 groups. Herein, there was no difference in the alpha-diversity of the intestinal microbiota between groups, whereas the results of beta-diversity analysis showed significant clustering according to dietary treatments, indicating that ileal microbiota community structure was altered by EO addition. Then, further analysis was performed on alteration of microbiota composition and specific taxa following EO addition. The enhanced digestive enzyme activity of ileum digesta in this study might be associated with the modulatory effects of EO on intestinal microbial composition. Actinobacteria, Bifidobacterials, *Deinococcus*, Bacillaceae and Caulobacteriales have been reported to be related to the improvement of animal feed utilization through producing extracellular enzymes (e.g., amylases and proteases) [45, 46]. Furthermore, Actinobacteria and Caulobacterales were characterized with their significant capacity of decomposing undigested components in feeds by secreting endogenous enzymes (cellulases, chitinases, xylanases, and pectinase) [47]. These enzymes can partially hydrolyze low-digestible components in poultry diets and mitigate the antinutritional effects by reducing the viscosity of gut digesta, thus improving nutrient digestion and absorption [5]. It was supported by the findings in this study that Bifidobacteriaceae (*Aeriscardovia*) and Caulobacteraceae (*Caulobacter*) were positively correlated with the activity of chymotrypsin and lipase. Therefore, the enrichment of Actinobacteria, Bifidobacterials, *Deinococcus*, Bacillaceae and Caulobacteriales in EO supplementation group could represent the promotion of endogenous digestive enzymes secretion, possibly favoring the increased activity of digestive enzymes and the improved feed utilization efficiency.

**Fig. 6:**
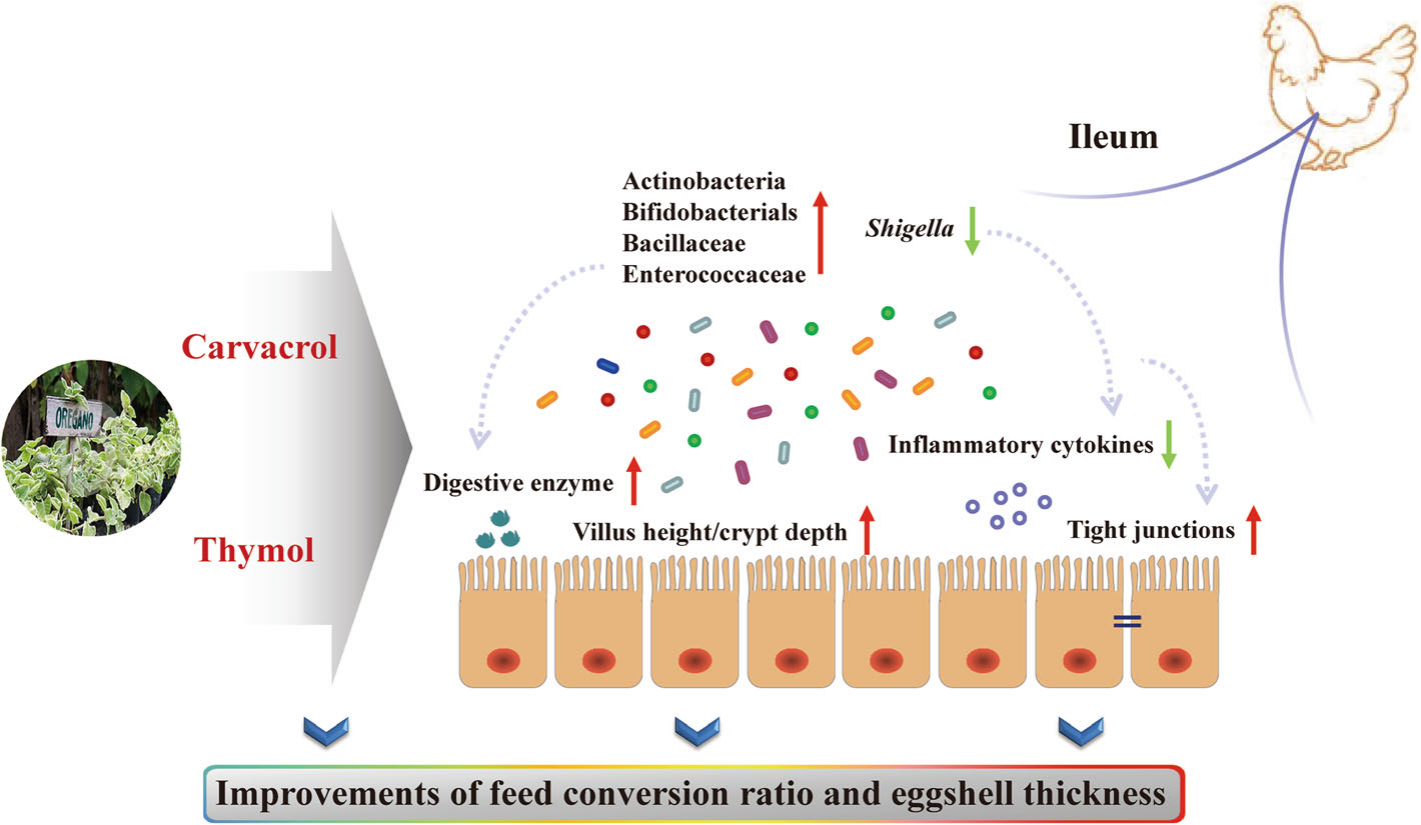
Proposed functions of essential oil in late-phase laying hens. Items with a red up-arrow indicated the increased bacteria, activity of digestive enzyme, villus height to crypt depth ratio or mucosal gene expression in the essential oil-supplemented group compared to the control, whereas those with a green down-arrow indicated the decreased ones in the essential oil-supplemented group

The antimicrobial activity of EO containing carvacrol or thymol has been widely tested against poultry pathogens such as *Escherichia-Shigella* [48], *Campylobacter* [49] and *Salmonella* spp. [50]. The antimicrobial mechanisms may be related to the perturbation of the lipid fraction in the plasma membrane, resulting in the altered membrane permeability and the leakage of intracellular materials of pathogen bacteria [51, 52]. In this study, dietary supplementation with EO inhibited the number of pathogens (*Shigella*) in the ileum compared to those in the control. *Shigella* was the core component of bacterial diarrhea in human and animals [53], and its invasion can cause physical impairment of tight junctions through direct interactions between secreted bacterial products and intestinal epithelial cells [54]. Numerous studies showed that the increased abundance of *Shigella* was closely associated with the poor animal production performance, the destroyed intestinal integrity and the raised levels of pro-inflammatory cytokines [55]. In addition, EO treatment could increase the abundances of some beneficial bacteria such as Actinobacteria, Bifidobacteriales, Enterococcaceae and Bacillaceae, which are helpful for the maintenance of overall microbial structure. Actinobacteria was regarded as keystone taxa to modulate the functionality of intestinal microbiota owing to the production of bacteriocins [46] and their ability to convert feedstuff into fermentable microbial biomass [56]. *Enterococcus*, *Bifidobacteria* and *Bacillus* have attracted great interests as natural antimicrobial probiotics to prevent diarrhea, improve feed efficiency and promote growth in animal production [57, 58]. They are capable to protect against potential pathogens by producing bacteriocins and blocking the adhesion of pathogens to the intestinal mucosa [59–61]. Thus, these results indicate that dietary EO inclusion may be beneficial for inhibiting pathogen colonization in the ileum of laying hens. In addition, *Bifidobacteria*, as the dominant microflora in normal intestine [62], could reinforce intestinal mucosal immune barrier functions by increasing the number of goblet cells and the secretion of mucin-2 [63]. Members of *Bacillus* are reported to participate in initiating and regulating immune responses by regulating cytokine expression [64] and showed positive association with the enhancement of intestinal tight junctions [46, 65]. Similarly, this study indicated that Bacillaceae abundance had a negative relationship with the expression of ileal pro-inflammatory cytokines (*IL-1β* and *IFN-γ*). Corynebacteriaceae may exert modulatory effects on inflammation responses, whose members like *Corynbecterium pyruviciproducens*, have been shown to stimulate the maturation and proliferation of dendritic cells and up-regulate Th2 responses in mice [66]. The functions of *Burkholderia* and *Kocuria* in inflammation responses were still unknown. However, an increase in gut *Burkholderia* abundance was accompanied by the improvements of intestinal structure and growth performance in chickens following a probiotic addition [67], and accompanied by the attenuation of plant extracts on *S. pullorum*-induced gut inflammation and the improvements of laying performance [68], implying a favorable effect of *Burkholderia* on gut health of chickens. Decreased population of *Kocuria* was one of the specific changes responsible for gut microbiota dysbiosis caused by *Salmonella enterica* serovar Infantis [69]. Conversely, the expansion of *Kocuria* was postulated to be responsible for decreased levels of proinflammatory cytokines *IL-6*, *8* and *IFN-γ* in neonatal piglets treated with sodium butyrate [70], indicating its contribution to alleviating intestinal inflammation. In this study, the suppressed abundance of pathogens as well as the increased abundance of some health-promoting bacteria might conduce to the improved gut morphology, enhanced epithelial barrier functions and the amelioration of intestinal inflammation in EO supplemented group (Fig. 6).

## Conclusions

In conclusion, this study demonstrated that dietary EO supplementation decreased feed efficiency and enhanced eggshell quality of late-phase laying hens by improving gut morphology, digestive enzyme activity, epithelial barrier functions and immune status, which could be in part responsible by the modulation of gut microbial profile. These findings may provide insights into the underlying mechanism of regulatory roles of EO on production performance and gut health in the late phase of production of laying hens.

## Data Availability

The sequencing datasets are available in the Sequence Read Archive of National Center for Biotechnology Information (accession number: PRJNA693086).

## Abbreviations

CD: Crypt depth
EO: Essential oil
FCR: Feed conversion ratio
IFN-γ: Interferon-γ
IL: Interleukin
LDA: Linear discriminant analysis
LEfSe: Linear discriminant analysis combined effect size measurements
PCoA: Principal coordinate analysis
TLR: Toll-like receptors
TNF-α: Tumor necrosis factor-α
VCR: Villus height to crypt depth ratio
VH: Villus height
ZO-1: Zonula occludens-1
